# Avian Community Responses to Variability in River Hydrology

**DOI:** 10.1371/journal.pone.0083221

**Published:** 2013-12-10

**Authors:** Alexander Royan, David M. Hannah, S. James Reynolds, David G. Noble, Jonathan P. Sadler

**Affiliations:** 1 School of Geography, Earth & Environmental Sciences, College of Life & Environmental Sciences, University of Birmingham, Edgbaston, Birmingham, United Kingdom; 2 Centre for Ornithology, School of Biosciences, College of Life & Environmental Sciences, University of Birmingham, Edgbaston, Birmingham, United Kingdom; 3 The British Trust for Ornithology, The Nunnery, Thetford, Norfolk, United Kingdom; University of Yamanashi, Japan

## Abstract

River flow is a major driver of morphological structure and community dynamics in riverine-floodplain ecosystems. Flow influences in-stream communities through changes in water velocity, depth, temperature, turbidity and nutrient fluxes, and perturbations in the organisation of lower trophic levels are cascaded through the food web, resulting in shifts in food availability for consumer species. River birds are sensitive to spatial and phenological mismatches with aquatic prey following flow disturbances; however, the role of flow as a determinant of riparian ecological structure remains poorly known. This knowledge is crucial to help to predict if, and how, riparian communities will be influenced by climate-induced changes in river flow characterised by more extreme high (i.e. flood) and/or low (i.e. drought) flow events. Here, we combine national-scale datasets of river bird surveys and river flow archives to understand how hydrological disturbance has affected the distribution of riparian species at higher trophic levels. Data were analysed for 71 river locations using a Generalized Additive Model framework and a model averaging procedure. Species had complex but biologically interpretable associations with hydrological indices, with species’ responses consistent with their ecology, indicating that hydrological-disturbance has implications for higher trophic levels in riparian food webs. Our quantitative analysis of river flow-bird relationships demonstrates the potential vulnerability of riparian species to the impacts of changing flow variability and represents an important contribution in helping to understand how bird communities might respond to a climate change-induced increase in the intensity of floods and droughts. Moreover, the success in relating parameters of river flow variability to species’ distributions highlights the need to include river flow data in climate change impact models of species’ distributions.

## Introduction

The physical and ecological structures of riverine-floodplain ecosystems are controlled by variability in river flows[1,2]. River flow influences in-stream ecological communities through changes in factors such as velocity, depth, water temperature, turbidity, channel stability and nutrient fluxes[1,2]. River flows interconnect in-channel, riparian and floodplain zone habitats to create an ecologically dynamic system[2] whereby flooding (inundation) has consequences on invertebrate communities and creates greater diversity and variability in functional traits[3,4]. The ability of invertebrates to tolerate high levels of inundation-driven pressure is determined by their resilience (i.e. ability to recover) and resistance to flow-induced disturbances[5], and by their ability to utilise habitat patches as refugia during high flows[6]. Disturbance in the organisation of lower trophic levels (e.g. primary producers such as phytoplankton) are conveyed through the food web and can result in reduced food availability to consumer species[7–9].

Climate change poses a severe threat to freshwater biodiversity as river flows are coupled closely to atmospheric drivers; thus, climate change will lead to the intensification of key processes in the global water cycle such as precipitation, runoff and evaporation, and shifts in drought and flood events[10,11]. Moreover, observations and models indicate that hydroclimatological variability is outside of ‘natural’ ranges already and consistent with anthropogenically-enhanced global warming[12]. More extreme and/or more frequent high and low flows will threaten aquatic communities by removing vulnerable taxa, and can result in a significant increase in the proportion of small-sized species[13] and a reconfiguration of biomass fluxes and food web structure[7]. Whilst this can lead to greater extinction for some predators, others may benefit from short-term increases in *r*-selected focal prey species that are able to exploit disturbance [7].

Although much less is known about riparian compared with in-stream dynamics, a few studies have shown that flow disturbances affect riparian invertebrate species’ assemblages and determine species’ interactions between trophic levels in food webs (e.g.^14^–^16^). As for in-stream environments, we hypothesise that riparian communities are influenced by river flows sensitive to climate change. However, our understanding of how variability in the river flow regime (e.g. flow magnitude, high and low flow variability, timing, frequency) shapes riparian species’ distribution and ecological structure is limited, particularly at higher trophic levels (e.g. tertiary consumers such as birds). Therefore, quantification of the relationships between river flow variability and riparian ecology is an urgent and important research challenge in the context of unravelling and projecting the impacts of climate change-induced flow alteration.

River birds represent an excellent focal taxon because river flow is a key predictor of patterns of species’ occurrence[17]. These species are often at the top of food chains and so are sensitive to disturbance at lower trophic levels including spatial and temporal mismatches in the availability of their prey[18,19], and pulses in flow may determine the timing of foraging[20] and breeding[21] behaviours. Regulation of river flows may influence the abundance[22], breeding success and survival[23] of river birds through modification of aquatic insect emergence and consequent prey availability[24]. Moreover, seasonal fluctuations in invertebrate prey fluxes from aquatic to terrestrial habitats subsidise the diets of river birds[25], resulting in dramatic shifts in aquatic prey use and foraging behaviour according to species-specific foraging tactics[26]. This may include a shift in species’ seasonal distributions whereby species move upland to take advantage of the post-breeding increase in terrestrial prey production relative to lowland aquatic production[22,25]. However, previous investigations of river flow-avian relationships are spatially and temporally constrained, with most focusing on a single watershed after a specific flood event. The influence of low flows (i.e. drought) on river birds is also not well researched (but see[27]). 

Here, we use data from a long-term bird monitoring scheme and river flow archives to investigate the relationship between avian species’ occurrence and river flow regime attributes across Great Britain. We selected *a priori* hydrological shifts which are consistent with those anticipated under climate change[12] to investigate the hypotheses that the probability of bird species’ occurrence is reduced for rivers characterised by greater hydrological fluctuations, including:

1 larger variability around high and low flows;
2 higher frequency of extreme flow events;
3 higher flow variability during species’ breeding seasons;
4 extreme high or low flow magnitude.

We discuss how species’ responses to these key attributes of river flow are mediated by life-history traits that influence their distribution in river-floodplain ecosystems.

**Figure 1:**
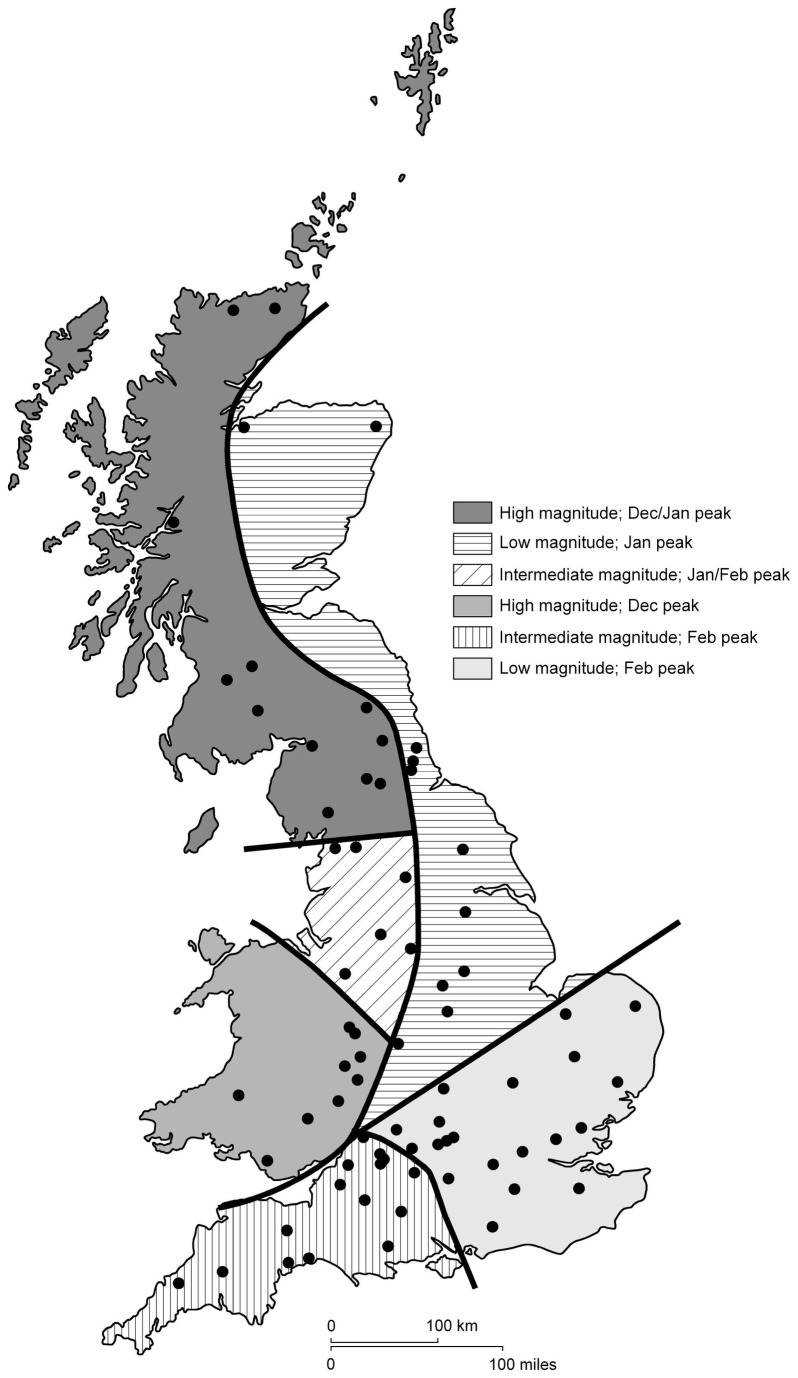
Distribution map of the 71 WBBS locations. Legend: Shaded areas on the map are indicative of the hydrological regions within which each survey site is located. Hydrological regions were determined on the basis of flow regime shape (based on timing of major runoff peaks) and flow regime magnitude (based on the mean, maximum, minimum, and standard deviation of average monthly flows[54]).

Presence/absence data for 17 river bird species were extracted from the British Trust for Ornithology’s (BTO’s) Waterways Breeding Birds Survey (WBBS) for 71 river locations, which captured a wide range of the hydrological variability across Great Britain (Figure 1). Bird data were paired with mean daily river flows from the UK National River Flow Archive (NRFA). The relationships between river bird occurrence and river hydrology were characterised using parameters that each quantified one of five hydrological facets: magnitude, frequency, high flow variability, low flow variability and timing (Table 1), within a Generalized Additive Model (GAM) framework. The relative importance of each hydrological parameter was assessed using an information-theoretic model averaging approach[28].

**Table 1:** Description of five hydrological indices used as predictors of the presence or absence of river bird species in Great Britain.

Predictor	Range	Description
***High flow variability***		
Three Day Maximum (m^3^)	1.549 - 83.607	Average annual 3-day maximum divided by median annual discharge. A measure of annual variability around high flows and the deviation of high flows from the median. High values imply greater variability in the magnitude of high flows and water depth while low values imply stability in high flows
***Low flow variability***		
Three Day Minimum (m^3^)	0.009 - 0.645	Average annual 3-day minimum divided by median annual discharge. A measure of annual variability around low flows and the deviation of low flows from the median. High values imply greater stability in the magnitude of low flows and water depth while low values imply variability in low flows
***Frequency***		
High Flow Frequency	0 – 124	Number of high flow days per year above three times the median. A measure of the number of extreme high flow days on a river during the UK hydrological year (October- September)
***Timing***		
April Flow Variation (m^3^)	0.011 - 73.657	Standard deviation of April discharge. A measure of flow variability during birds’ breeding seasons. High values imply greater flow variability while low values imply stability in flows during birds’ breeding seasons
***Magnitude***		
Mean Daily Flow (m^3^)	0.223 - 117.812	Mean value of daily discharge divided by median of daily discharge. A measure of flow magnitude providing an estimate of river size

## Results

**Figure 2:**
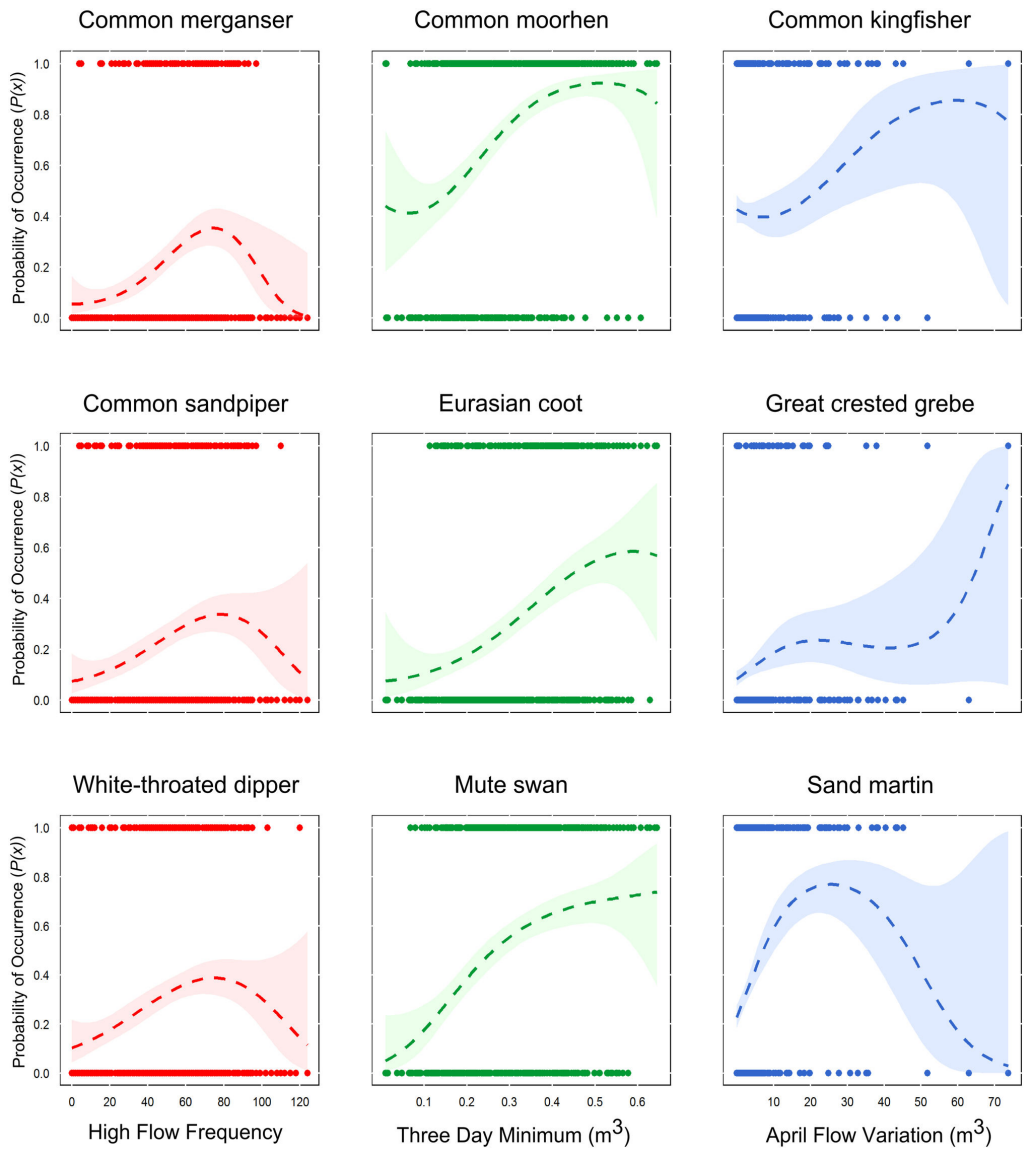
Examples of non-linear relationships from models between species’ *P*(*x*) and three measures of hydrological variability. Legend: High Flow Frequency (red), a measure of the number of extreme high flow days; Three Day Minimum (green), a measure of low flow variability and the deviation of low flows from the median; and April Flow Variation (blue), a measure of flow variability during the species’ breeding seasons. Dashed lines give the probability curve from a GAM with a cubic regression spline and two degrees of freedom. Shaded regions represent 95% confidence limit for the spline fit.

**Figure 3:**
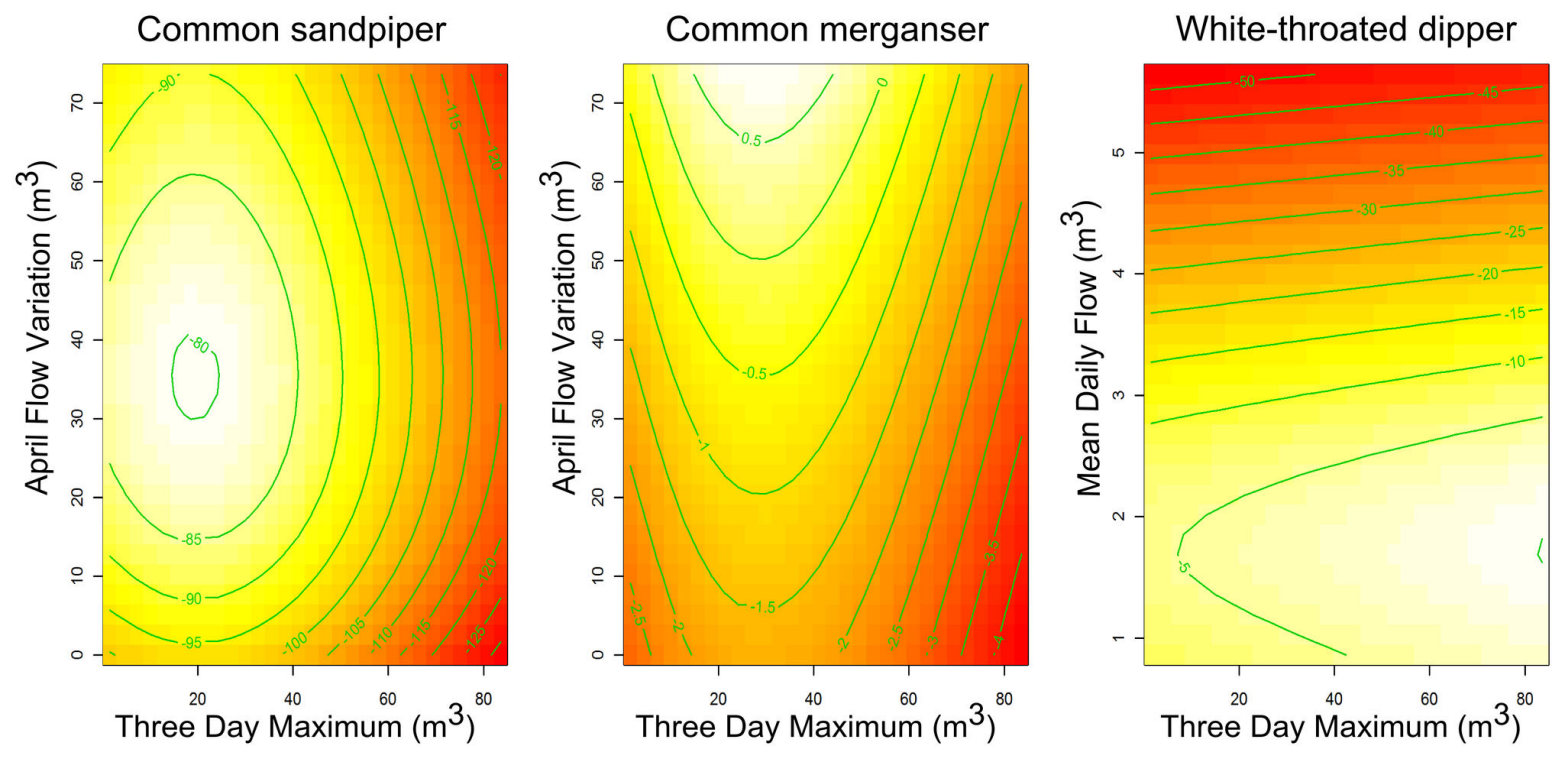
Example response surfaces showing the relationship between species’ *P*(*x*) and two hydrological indices. Legend: Surface plots showing the interactive effects of two hydrological indices on species’ *P*(*x*), where lighter areas of shading illustrate a stronger influence on *P*(*x*). When considered independently, the indices have limited effects on *P*(*x*) compared to the combined effect of both indices.

All 17 focal bird species had a strong association with at least one of the five hydrological indices with selection probabilities (*Sps*) well above that of the null predictor interval (Table 2). Model performance was assessed using the area under the curve (AUC) of a receiver operating characteristic (ROC) plot (an indicator of the goodness-of-fit of the model that is independent of the threshold probability at which the presence of the target organism is accepted) and Cohen’s Kappa (*K*) (measures the level of agreement between observed occurrences and absences with those predicted by the model after accounting for chance effects). According to AUC, nine species’ model sets had ‘high accuracy’ (i.e. AUCs > 0.9) and seven were ‘useful’ (i.e. 0.7 < AUCs ≤ 0.9)[29] (Table 2). According to *K*, the strength of agreement between model predicted values (values set as: absences < 0.5 < presences) and observed response values varied from ‘almost perfect’ (0.81 < Ks ≤ 1) (common sandpiper [*Actitis hypoleucos*], Eurasian oystercatcher [*Haematopus ostralegus*], white-throated dipper [*Cinclus cinclus*]) to ‘slight’ (0 < Ks ≤ 0.2) (grey heron [*Ardea cinerea*], western yellow wagtail [*Motacilla flava*])[30]. Species’ models broadly support hypotheses 1-4, but with important and clear differences in hydrological associations observed between species (Figures 2 and 3) that were consistent with their respective life-history traits.

**Table 2:** Selection probabilities (*Sps*) of five hydrological indices for 17 river bird species in Great Britain.

**Species**	**Three Day Maximum**	**Three Day Minimum**	**High Flow Frequency**	**April Flow Variation**	**Mean Daily Flow**	**Null**	**Models < ∆AIC 2**	***K***	**AUC**
Common kingfisher *Alcedo atthis*	0.535(±)	0.402	0.391	0.998(+)	0.312	0.345-0.382	7	0.261	0.738
Common merganser *Mergus merganser*	0.533(±)	0.318	0.467(±)	0.858(+)	0.415	0.272-0.341	9	0.499	0.910
Common moorhen *Gallinula chloropus*	0.285	0.999(+)	0.295	0.377	0.915(-)	0.393-0.426	4	0.591	0.890
Common reed bunting *Emberiza schoeniclus*	0.669(-)	0.303	0.731(-)	0.860(+)	0.819(+)	0.342-0.405	4	0.507	0.875
Common sandpiper *Actitis hypoleucos*	0.995(±)	0.439	0.982(±)	0.999(±)	0.360	0.499-0.500	4	0.891	0.994
Eurasian coot *Fulica atra*	0.369	0.945(+)	0.718(-)	0.361	0.469	0.378-0.427	6	0.582	0.904
Eurasian oystercatcher *Haematopus ostralegus*	0.491	0.932(-)	0.999(-)	0.019	0.992(-)	0.366-0.429	2	0.963	0.998
Eurasian reed warbler *Acrocephalus scirpaceus*	0.289	0.625(+)	0.180	0.453	0.423	0.379-0.454	7	0.496	0.916
Great cormorant *Phalacrocorax carbo*	0.693(+)	0.949(+)	0.285	0.999(+)	0.546(-)	0.366-0.432	4	0.377	0.822
Great crested grebe *Podiceps cristatus*	0.295	0.330	0.307	0.999(±)	0.360	0.337-0.429	5	0.533	0.945
Grey heron *Ardea cinerea*	0.299	0.997(+)	0.378	0.440	0.410	0.334-0.382	7	0.162	0.694
Grey wagtail *Motacilla cinerea*	0.568(+)	0.408	0.800(±)	0.354	0.652(+)	0.385-0.441	10	0.488	0.838
Mute swan *Cygnus olor*	0.286	0.999(+)	0.551(-)	0.999(±)	0.327	0.331-0.430	5	0.617	0.918
Northern lapwing *Vanellus vanellus*	0.319	0.989(-)	0.347	0.303	0.828(-)	0.372-0.443	3	0.581	0.901
Sand martin *Riparia riparia*	0.341	0.320	0.386	0.999(±)	0.353	0.336-0.412	5	0.526	0.891
Western yellow wagtail *Motacilla flava*	0.549(-)	0.801(+)	0.960(-)	0.385	0.706(-)	0.330-0.429	5	0.213	0.877
White-throated dipper *Cinclus cinclus*	0.947(+)	0.620(-)	0.967(±)	0.596(±)	0.999(-)	0.425-0.465	3	0.868	0.986

*Sps* were calculated by summing Akaike weights (AIC*w*_*i*_) of all model permutations containing each predictor. Parameters included in a greater proportion of the best-supported models have larger *Sps*, considerably above the null predictor, thereby demonstrating strong support for their inclusion in the best approximating model. The inclusion of parameters with lower *Sps* is less important for obtaining good model fit. The null interval was calculated from simulations of 100 randomly generated predictors, summing AIC*w*_*i*_s of all models containing each null predictor and then using the highest 10 values to calculate 95% confidence intervals. For high *Sps*, (+) indicates a positive relationship, (-) a negative relationship and (±) a quadratic relationship (see figures 2-3 for graphical representation). The number of models with Akaike Information Criteria (AICs) within two of the best fitting model is also given to provide an estimate of uncertainty around specification of the best approximating model. The average *K* and AUC for this reduced model set are provided as measures of model performance. n=574 survey years from 71 WBBS locations.

Species associated with upland environments (e.g. common merganser [*Mergus merganser*], common sandpiper, grey wagtail [*Motacilla cinerea*], white-throated dipper) displayed positive and quadratic relationships with Three Day Maximum, which suggests their probability of occurrence (*P(x*)) increases with higher flows. For these same upland species, quadratic associations were observed with High Flow Frequency, indicating increased *P*(*x*) under more high flow disturbance (Figure 2). Ten species had either positive or negative associations with Three Day Minimum; species that forage typically within aquatic macrophytes (e.g. common moorhen [*Gallinula chloropus*], Eurasian coot [*Fulica atra*], grey heron and mute swan [*Cygnus olor*]) preferred stability around low flows (Figure 2), whereas some species that forage predominantly at terrestrial river margins (e.g. Eurasian oystercatcher, northern lapwing [*Vanellus vanellus*]) benefited from increased low flow variability. White-throated dipper, which typically feeds in the river channel, also favoured increased low flow variability which may indicate a propensity for this species to forage opportunistically outside breeding territories when marginal habitats are exposed.

Flow timing was an important predictor of occurrence for nine species. In particular, April Flow Variation was the most important predictor of occurrence for the three diving species (i.e. common merganser, great cormorant [*Phalacrocorax carbo*], great crested grebe [*Podiceps cristatus*]) and the two bank-nesting species (i.e. common kingfisher [*Alcedo atthis*], sand martin [*Riparia riparia*], Figure 2). Interpretation of species’ relationships with this parameter, however, is somewhat hindered by a lack of data at higher discharges, which is reflected by the larger confidence intervals around the smoothed line in Figure 2. Species displayed largely negative associations with Mean Daily Flow, although both common reed bunting (*Emberiza schoeniclus*) and grey wagtail had positive associations. 

## Discussion

This study has demonstrated that the occurrence of river birds is influenced strongly by elements of river flow variability. By quantifying the different facets of flow regime, we have shown that species’ distributions are characterised by complex responses to: (i) variability around extremes of high and low flows, (ii) flow frequency, (iii) the timing of flow events, and (iv) flow magnitude. It is well established that river flows and hydrological variability influence the distribution and trajectories of the life history of aquatic and, to a lesser extent, riparian invertebrates; however, this quantitative study provides evidence of the influence of river flows on species’ distributions extending beyond lower trophic levels to tertiary consumers at the top of aquatic/riparian food webs. Fluctuations in flow conditions may impact these species more heavily primarily by (a) causing disturbances in aquatic food webs, which decrease prey abundance or shift prey composition, and (b) temporarily altering the availability of foraging and breeding habitats.

Altered river flow regimes may impact riparian species at higher trophic levels through the removal of prey adapted to life in aquatic habitats under specific flow conditions, which may lead to a breakdown in energy fluxes, removal of trophic pathways and compromised food web resilience[7–9,22–25]. Variability in high flows causes large fluctuations in water velocity and depth, which can determine the availability of foraging habitats[20] and influence foraging efficiency and net energy gain[31]. We found clear positive quadratic associations between the distribution of some species and measures of high flow variability and high flow frequency, suggesting that some species require a range of variability around high flows. Intermediate measures of high flow may provide optimal foraging habitat for some riparian fauna whereas floods may decrease prey abundance and shift prey composition[18,19], perhaps resulting in increased consumer competition or broadening of foraging niches, while exceptionally large or prolonged extreme flow events may lead to marked increases in adult and juvenile mortality for the most sedentary of species[18].

Fluctuation of low flows increases heterogeneity in the spatial and temporal extent of river marginal habitat[1,2]. We found clear positive associations between the occurrence of some species that forage predominantly at river margins and measures of low flow variability. This suggests low flow variability positively influences the availability or prevalence of foraging habitats for these riparian consumer species. However, species that forage or breed within macrophytes in-stream or at river margins preferred stability around low flows, perhaps because substrates remain undisturbed promoting greater diversity and growth of these plants[32].

Changes in the timing of flows may be attributed to climate change[12]. Riparian species can be adversely affected by increased flow variability during sensitive periods of their annual cycles such as during breeding, resulting in reproductive failure[23,33], increased dispersal[34] or increased adult mortality[18]. We found the occurrence of bank-nesting species (i.e. common kingfisher, sand martin) was sensitive to flow timing. Bank-nesting species may be particularly vulnerable to flow variability during breeding seasons as nest sites on marginal habitats are prone to inundation (e.g.[34]). Both species nest in exposed river sediment banks that are formed during scouring high flows, suggesting annual variability in high flows across seasons is required for the creation of breeding habitat. The greater tolerance of breeding season flow variability exhibited by the common kingfisher models may reflect a propensity for this solitary bank-nester to nest more frequently on tributaries, where it is buffered from the impact of the highest flows on the main channel. As such, the vulnerability of riparian species to flow variability during sensitive periods of their annual cycle may be determined by a combination of the sensitivity and plasticity of intrinsic behavioral traits, such as nest site selection [35].

River systems are vulnerable to climate change and current hydrological simulations in regional climate models for the UK predict that by 2050 river flows will have changed considerably, with models broadly predicting decreases in summer flows and increases in winter flows[36]. Our results indicate that future ecological consequences of changes in river flow are not restricted to aquatic communities but may have profound effects on other riparian biota such as birds. It is well established that climate change may increase the extinction risk and strongly influence the phenology and dynamics of bird populations[37,38]. Climate models can be used to predict future changes in bird species’ ranges based on air temperature shifts; however, such models for bird species’ distribution do not take into consideration river flow variability[39] and rarely even account for changes in habitat. Our findings have implications for climate change impact models as they emphasise the need to include the effects of hydrological change on riparian biota, as well as the value of using long-term, spatially-extensive datasets to understand flow variability, including the importance of extreme events. 

Incorporating flow variables into such models has the potential to improve future projections beyond those based on climate alone as they may identify areas that will become unsuitable owing to non-climatic factors and prevent over-prediction of climate change impacts[40]. Moreover, the incorporation of flow parameters into models to describe climate-driven changes in species’ habitat represents a more biologically realistic approach because they include small-scale habitat attributes overlooked by coarse large-scale climate models[41]. Interpreting the future distributions of river birds requires new research to assess the ecological consequences of climate and hydrological extremes on aquatic and riparian ecosystems. More fundamentally, conservation assessments are reliant on longitudinal long-term surveys, which facilitate the detection and monitoring of temporal and spatial patterns in river bird populations. 

The success in relating hydrological indices to the distributions of river birds in this study demonstrates that variability in river flow regime has consequences for the distribution of riparian species and ecological structure at high trophic levels in aquatic-riparian food networks. By using national-level, long-term datasets, we were able to identify spatial and temporal patterns in species’ relationships with the hydrological indices. Species’ occurrence changed with variability in both high and low flows, including the frequency of extreme flow events, as well as with variability in both the timings and magnitude of flows. These relationships were complex but could be explained by ecological traits that characterise species within aquatic-riparian ecosystems. This paper represents an important contribution in helping to understand how bird communities might respond to a climate change-induced shift in river flow and also highlights a potential vulnerability of species to an increase in the intensity of floods and droughts. We believe that our approach will not only generate new insights as reported here but also establish foundations for further work on modelling of the impact of river flow variability on both avian and non-avian water-dependent taxa. 

## Materials and Methods

WBBS bird data between 1998 and 2010 inclusive were used, although data from year 2001 were excluded from analyses as a very small proportion of locations was surveyed due to the foot-and-mouth outbreak; this required large-scale quarantine measures to limit the spread of disease and thereby restricted access to the countryside. Each survey location was surveyed during at least three years during the survey period and at least once since 2008. Each location comprised a single stretch of river averaging 3 km in length (range of 0.5 to 5 km) at least partly overlapping a focal randomly selected 2 × 2 km tetrad. BTO WBBS guidelines state that permission to access field sites is gained from relevant landowners prior to the commencement of studies. WBBS surveyors record all bird species but the number of species used in the analysis was constrained by their occurrence across the sample sites as, to reduce model instability, we only analysed species for which records comprised no less than 10% of the response values; the least prevalent species was the western yellow wagtail which occurred in 10.3% of site-year combinations (mean prevalence of all species used was 37.7%). Individual years at each site were treated separately, totalling 574 separate site year combinations. Presence/absence data per stretch of river were extracted for each year separately. Mean daily river discharges were used from gauging stations within 10 km of each WBBS sample location. We did not analyse paired data where a major tributary inflow occurred between the gauging station and survey location. For gauging stations with < 10% missing values for any one year, we interpolated data gaps using long-term mean daily flows[42]. Gauging stations with ≥ 10% missing values were excluded from the analysis. The hydrological year in the UK runs from October to September[43] and bird data were paired with hydrological data from the associated hydrological year (e.g. bird data from spring 2010 were paired with hydrological indices calculated from daily flow data between 1^st^ October 2009 and 30^th^ September 2010). Thus, hydrological variability was measured before, during and after the birds’ breeding seasons. 

As there is concern that considerable multi-collinearity exists amongst many widely used hydrological indices[44,45], we considered our model parameters *a priori*, whilst ensuring that each one was statistically independent, by producing multi-panel scatterplots[46], and had Variance Inflation Factor (VIF) scores below two[47]. We produced separate binomial GAMs, specifying a logarithmic link function, for all 17 species to quantify the relationship between the hydrological indices and species’ *P*(*x*). GAMs are a particularly useful regression method for species’ distribution modelling[48] as they do not force a parametric relationship between the response and predictor, and smoothers can be used to model complex non-linear relationships that are frequently observed in ecology. Where non-linear relationships were observed, a cubic smoothing spline was fitted to the predictor, with a fixed degree of smoothing (two degrees of freedom) so as to capture the trends in the data with the least number of degrees of freedom whilst preventing over-fitting[49]. GAMs were fitted using version 1.7-24 of the mgcv package for R[49]. Our response variable was defined as the presence or absence (i.e. non-detection) of a species during the survey in any one year, as specified by WBBS methodology (i.e. two visits per breeding season). To account for correlation between survey years and variation in the geographic coverage of WBBS sites, a three-way interaction between year, latitude and longitude was included as a fixed effect in all models. This approach controls for: (i) similarities in the response variable at nearby points by fitting a smooth two-dimensional surface to these data and (ii) unmeasured variables that may affect the response by fitting to response peaks and troughs, thereby fitting spatial autocorrelation in the dataset by optimising the degrees of freedom[50]. Additionally, the inverse of the number of transects completed at each location was included as an offset to account for variation in survey effort and corresponding probability of species’ detection. 

We utilised the Information-Theoretic (IT) model averaging approach for data analysis[28] as it corrects for potential model selection bias and error associated with parameter estimation and presents the results in the context of strength of evidence[28,51,52]. A model was produced for every permutation of predictors, resulting in 31 models per species, and the fit of each model was assessed using the Akaike Information Criterion (AIC). Akaike weights (AIC*w*_*i*_s) were calculated for all models as follows: 

$$\omega i=\frac{\exp(-\frac{1}{2}\Delta AICi)}{\sum_{r=1}^{n}\exp(-\frac{1}{2}\Delta AICr)}$$

where *w*_*i*_ is the probability that model *i* would again be selected as the model of best fit if the data were collected again under the same circumstances[28]. For all models *w*_*i*_ sums to 1, and *Sps* for each predictor were calculated by summing *w*_*i*_s for every model containing each predictor. Poor predictors do not always have *Sps* close to zero so we provided an approximate, yet conservative, *w*_*i*_ interval against which the importance of predictors was evaluated[53]. One hundred randomly generated predictors with a distribution between zero and one were produced and every model was run in turn with each of these. *Sps* were calculated for each null predictor and a 95% confidence null interval computed. Rather than use the *Sps* from all null predictors to produce the null interval (e.g. 53), we used just the 10 largest values as this produced a more conservative and robust interval against which *Sps* for the hydrological parameters could be compared. Only strong predictors of species’ occurrence should have *Sps* larger than this null interval. 
